# Laser acupuncture for refractory coccydynia after traumatic coccyx fracture: A case report

**DOI:** 10.1097/MD.0000000000018860

**Published:** 2020-02-07

**Authors:** Chien-Hung Lin, Szu-Ying Wu, Wen-Long Hu, Chia-Hung Hung, Yu-Chiang Hung, Chun-En Aurea Kuo

**Affiliations:** a Department of Traditional Medicine, Kaohsiung Chang Gung Memorial Hospital; b Department of Sports Medicine; c College of Medicine, Kaohsiung Medical University; d College of Nursing, Fooyin University, Kaohsiung; e Department of Orthopaedics, Fu Jen Catholic University Hospital, Taipei; f School of Chinese Medicine for Post Baccalaureate, I-Shou University, Kaohsiung; g Department of Nursing, Meiho University, Pingtung, Taiwan.

**Keywords:** bone healing, coccydynia, coccyx fracture, laser acupuncture

## Abstract

**Rationale:**

Coccyx fracture is an injury usually caused by trauma. In most cases, the fractures recover after conservative therapy. For refractory cases that exhibit coccydynia after more than 2 months of conservative treatment, coccygectomy is indicated. However, limited information about the efficacy of this procedure is available, and it is known to have a high complication rate. As such, other therapeutic approaches are needed. Here, we report our experience using another conservative treatment option, low-level laser therapy, to successfully reduce refractory coccydynia in a patient with coccyx fracture.

**Patient concerns:**

A 23-year-old woman had refractory coccydynia and increased pain after a traffic accident-induced coccyx fracture.

**Diagnoses:**

Initially, the patient reported transient improvement after conservative treatment with non-steroidal anti-inflammatory drugs. However, the pain increased in severity (numerical rating scale score of 8) soon after she resumed work in her office, and progressed in the following 2 months. Surgical intervention was suggested owing to the prolonged coccydynia following the failure of conservative treatment and difficulties in performing daily life activities. However, she sought other conservative therapy options, because she was concerned about the risks associated with the coccygectomy surgery.

**Interventions:**

The patient received low-level laser therapy once a week, for 24 weeks.

**Outcomes:**

After 11 weeks of treatment, the patient reported significant improvements in her symptoms; her pain was reduced to a numerical rating scale score of 2 and bone healing was noted on radiographs. The patient could eventually perform her daily activities satisfactorily, without coccydynia, after 24 weeks of treatment.

**Lessons:**

Laser acupuncture produced analgesic effects in this patient with refractory coccydynia after traumatic coccyx fracture. This is the first case report to apply laser acupuncture for refractory coccydynia after traumatic coccyx fracture. Our findings imply that laser acupuncture may be a good conservative therapy option for coccyx fracture.

## 1 Introduction

Coccyx fractures are usually caused by trauma, such as a backwards fall or childbirth, which may cause coccyx pain and interfere with sitting for long periods, sexual intercourse, or defecation.^[1,2]^ Generally, the diagnosis is made clinically, and lateral-view radiographs may show a displacement fracture.^[3]^ Treatments for coccyx fractures tend to be conservative, such as donut cushions, correcting the sitting posture, cold packing, hot bathing, or analgesic drugs, and produce good outcomes in most cases; however, surgery is an option if coccydynia, that is, pain in the region of the coccyx, persists for over 2 months.^[2,3]^ Although coccygectomy is indicated for patients with refractory coccydynia, there is limited data about the efficacy of this procedure and the surgery is known to have a high complication rate.^[1]^ Thus, other conservative treatment options for refractory coccydynia are needed.

Low-level laser therapy (LLLT), or laser acupuncture, is used for many clinical conditions, and has been well accepted as a useful treatment option because it is non-invasive and is a pain-free process. In addition, laser acupuncture has been shown to have anti-inflammatory effects and to promote tissue healing, thus alleviating acute or chronic pain.^[4,5]^ A systematic review published in 2018 that investigated the bone healing effects of LLLT reported that both animal studies and human clinical research supported the potential utility of this approach for bone healing^[6]^; however, few reports examining the ability of LLLT to reduce pain in fracture patients exist.

In this report, we describe our experience using LLLT as the main treatment modality for a patient with coccyx fracture with refractory coccydynia 6 weeks after being injured in the traffic accident (August 14, 2016). She presented with progressive pain and visited our clinic on December 14, 2016. Bone healing was noted on the radiograph after 11 weeks of treatment. The patient's symptoms improved much after 24 weeks of LLLT treatment and she was able to perform her daily activities well without coccydynia. Our findings imply that LLLT may be another conservative strategy for pain management and bone healing in patients with coccyx fracture.

## 2 Case presentation

The patient has provided informed consent for publication of the case, and the study design was approved by the Chang Gung Medical Foundation Institutional Review Board (IRB No. 201701034B0).

A 23-year-old woman, with no past medical, traumatic, or surgical history, complained of coccyx and low-back pain after a motor vehicle accident that occurred on August 14, 2016. Her motorcycle collided with another motorcycle, causing her to fall off, with her buttocks hitting the ground. Subsequently, she was admitted to one medical center in Kaohsiung. In the emergency room, she was unable to sit or lie down with her caudal vertebral area contacting the bed. She reported experiencing a sharp pain, which she rated a numerical rating scale (NRS) score of 8 at that time. The coccydynia also prevented her from walking or shifting from a supine to a seated position. The pain increased on contact with the caudal vertebrae, such as in the seated or supine positions.

**Figure 1:**
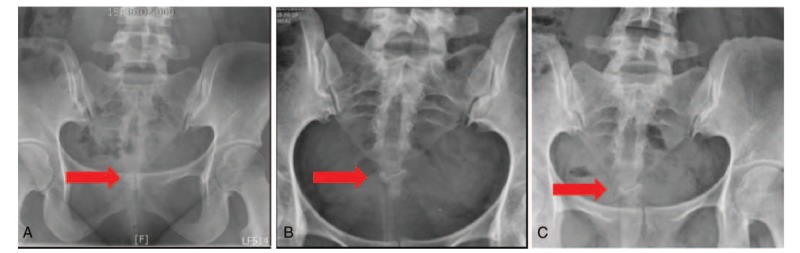
Anterior-posterior pelvic radiograph of the fracture at onset and post-therapy. (A) Image acquired immediately after the accident. Fracture dislocation of the sacrococcygeal joint (arrow) was noted after the traffic accident, which occurred on August 14, 2016. (B) Anterior-posterior pelvic radiograph acquired after 11 weeks of treatment, on March 1, 2017. The radiograph shows coccygeal healing (arrow). (C) Anterior-posterior pelvic radiograph acquired after 24 weeks of treatment, on May 31, 2017, shows coccygeal healing (arrow).

**Figure 2:**
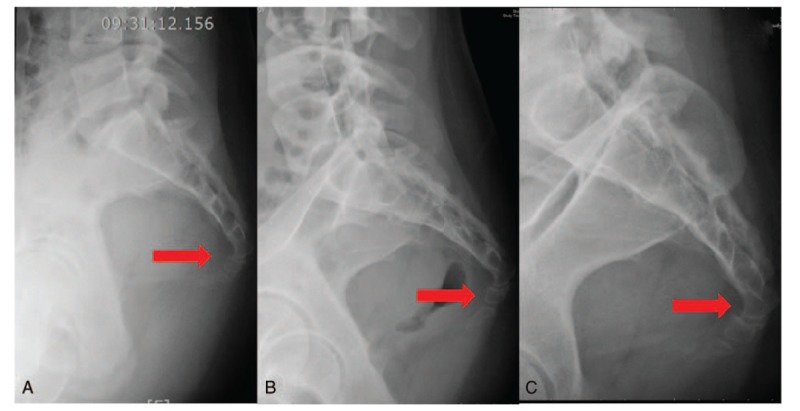
Lateral pelvic radiograph of the fracture at onset and post-therapy. (A) Image acquired immediately after the accident, which occurred on August 14, 2016. The lateral view of the sacrococcygeal joint shows joint deformity after trauma (arrow). (B) Lateral pelvic radiograph acquired after 11 weeks of treatment. This film was taken on March 1, 2017, at which point the clinical symptoms and numerical rating scale score had improved significantly. Bone healing was progressing without further dislocation (arrow). (C) Lateral pelvic radiograph acquired after 24 weeks of treatment. This image was obtained on May 31, 2017, and at this point, the patient was able to perform her daily activities well, without coccydynia.

A radiograph taken at the emergency department on August 14, 2016, showed fracture dislocation of the sacrococcygeal joint (Figs. 1A and 2A). The patient initially received non-steroidal anti-inflammatory drugs for pain control, and the coccydynia gradually improved to an NRS score of 5 after the conservative treatment. However, the pain increased 6 weeks after the accident when she resumed work, where she had to move heavy goods and sit for long periods of time. She continued to receive conservative treatment with oral analgesics. However, the pain persisted and prevented her from sitting for more than 5 minutes at a time. The NRS score increased from 5 to 8 in the subsequent 10 weeks. The orthopedic doctor recommended surgery, since the conservative treatment failed to reduce her pain; however, the patient opted for other conservative therapy and visited our clinic for acupuncture on December 14, 2016.

During the physical examination, direct visual inspection of the skin over the coccygeal region revealed mild protrusion of the coccyx. Sacrococcygeal palpation and the application of pressure over the sacrococcygeal junction and coccyx showed that the tenderness was localized to the side of the bone protrusion. Palpation of other non-coccygeal lumbosacral structures, including the pelvic bone, hip joints, and gluteal muscles did not reveal any specific findings. Neurologic examinations showed no obvious radiculopathy, and no decrease in lower-extremity muscle power was observed. However, because of the pain, the patient showed a weakened ability to perform the buttock squeezing exercise, which involves the gluteus maximus, gluteus minimus, and gluteus medius.

**Figure 3:**
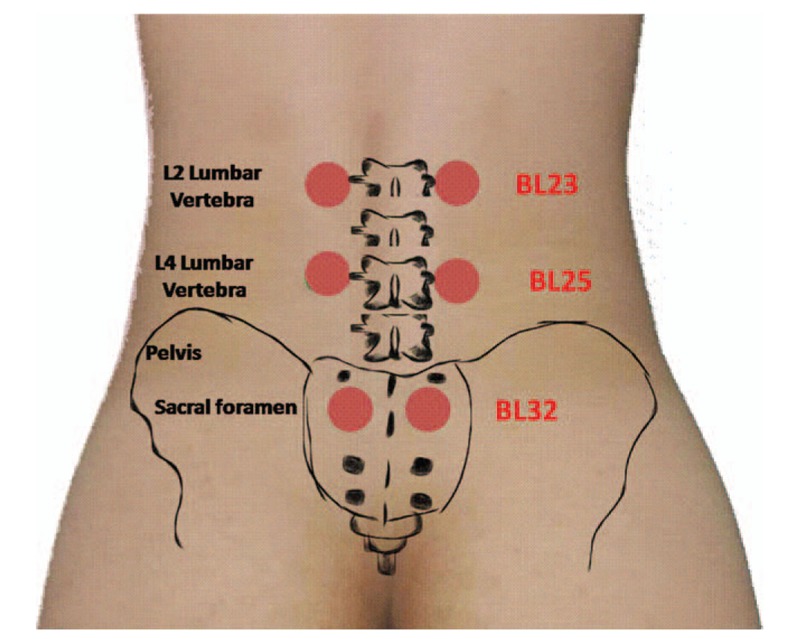
The locations of the BL23, BL25, and BL32 acupoints. BL23 is at the level of the lumbar 2 vertebra and 1.5 cun lateral to the lumbar 2 vertebra. BL25 is at the level of the lumbar 4 vertebra and 1.5 cun lateral to the lumbar 4 vertebra. BL32 is at the level of the sacral 2 foramen.

**Figure 4:**
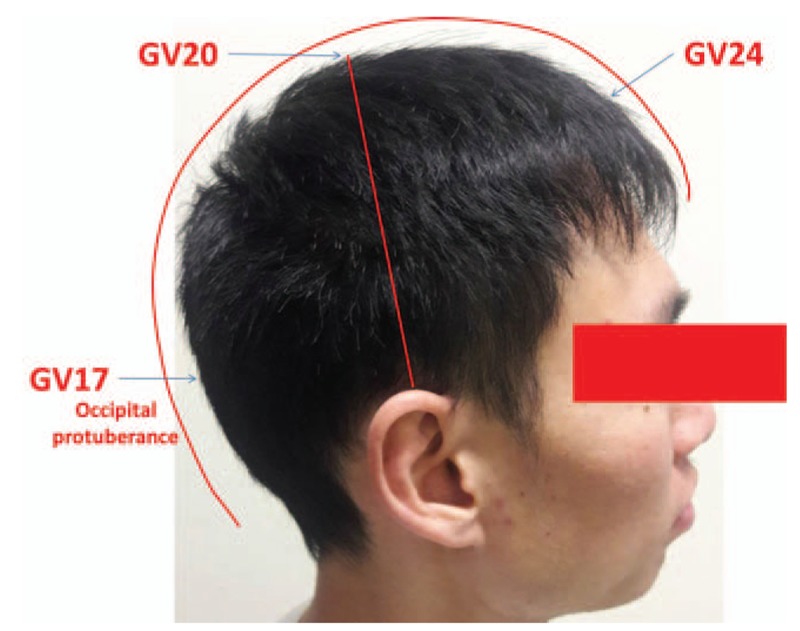
The location of the GV20 acupoint. The GV20 acupoint is positioned at the intersection of the midline and the line connecting the highest points of both ears.

On the basis of the patient's symptoms, we used laser acupuncture (RJ LaserPen 551A, REIMERS & JANSSEN GmbH, Germany; gallium aluminium arsenide 200-mW laser, 810 nm, 5 W/cm^2^, pulsed wave) (Table 1) at the BL23, BL25, and BL32 acupoints (frequency selection: Bahr 2, application time: 20 s per acupoint) (Fig. 3), the GV20 acupoint (frequency selection: Bahr 4, application time: 20 s per acupoint) (Fig. 4), and the ashi point (frequency selection: Nogier C, application time: 40 s per point).

**Table 1:**
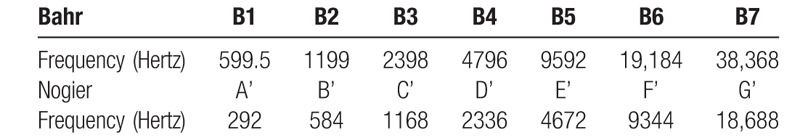
The frequencies corresponding to the mode settings of the RJ Laser Pen device used for performing laser acupuncture in our patient with coccyx fracture.

**Figure 5:**
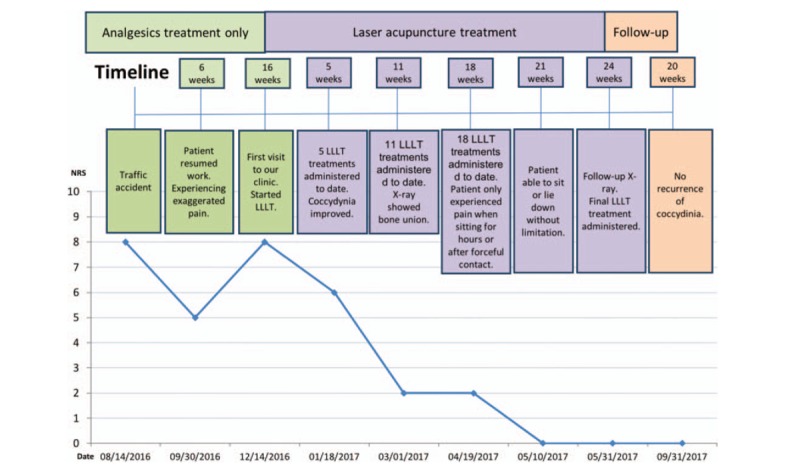
Numerical rating scale (NRS) scores reported by the patient over the course of treatment. The NRS score was 8 immediately after the traffic accident. The coccydynia improved initially after conservative treatment. However, the pain worsened after she returned to work. The NRS score increased to 8 at 6 weeks after the accident. The NRS score decreased gradually after the initiation of low-level laser therapy. The NRS decreased to 6 and 2 after 5 and 11 therapy sessions, respectively. Finally, she was able to perform her daily activities well, without coccydynia, after 21 weeks of treatment, at which point the NRS score was 0. CHP = Chinese herbal product; OPD = outpatient department.

The patient was only able to receive treatment once a week because of her work commitments. She did not receive any analgesic medications after LLLT was initiated. After five sessions of laser acupuncture (5 weeks after the initiation of LLLT treatment), her coccydynia and backache improved. She was able to sit and lie on her back for about 30 minutes, and the NRS score was 6. She continued to receive the same laser acupuncture regimen, and after 11 therapeutic laser acupuncture sessions (March 1, 2017), the patient was able to sit and lie on her back for about an hour, and the NRS score was 2. At the same time, a radiograph showed improved healing of the coccygeal bone (Figs. 1B and 2B). Although the lateral-view radiograph showed that some sacrococcygeal joint deformity remained, the patient reported significant improvements in her clinical symptoms. Eighteen weeks from the initiation of LLLT treatment (April 19, 2017), pain decreased even more, only happened when sitting for hours or strong direct contact on coccyx, with the NRS 2. Twenty-one weeks from the initiation of LLLT treatment (May 10, 2017), she did not feel tenderness in those occasions like sitting for hours or strong direct contact on coccyx. After 24 weeks of treatment (May 31, 2017), she was able to perform her daily activities well, without coccydynia, and the NRS score was 0 (Figs. 1C and 2C). She was able to sit on chairs with hard surface, carry out her regular work without difficulties, and even go out traveling and sitting in the car for hours. After full discussion, she received the last LLLT therapy and ended the treatment. During the 24 weeks of treatment, the patient was assessed with history taking and physical examination during every clinic visits, and she was able to mention about her symptom in the recent week. According to the CARE guideline, the intervention adherence and tolerability were appropriate. She attended the treatment every week and stated no pain, hematoma, or local irritation during or after each treatment. There was no loss of follow-up during the 6-month treatment period and another assessment was made 4 months after the end of the treatment (2017/09/30). According to the patient, the pain did not recur during the treatment-free period. The NRS scores obtained over the clinical course are presented in Figure 5.

## 3 Discussion

This report describes the successful use of laser acupuncture for pain management, with bone healing, in a patient with coccyx fracture. Our patient was concerned about the pain and possible complications associated with surgical intervention. Therefore, we opted to use laser acupuncture instead of surgery. Laser acupuncture is a non-invasive, painless procedure. No adverse effects were reported by the patient or observed by the physician. Following treatment, she was able to return to work and resume her regular daily activities. Despite the previous refractory coccydynia before she received LLLT treatment, which prevented her from performing some work functions, the NRS score, as reported by the patient, after 21 weeks of treatment was 0. The treatment response lasted after 4 months from the end of the treatment (the last interview timepoint). To our knowledge, this is the first case report to show that LLLT for fracture associated refractory coccydynia produces beneficial analgesic effects and bone healing.

Conservative treatment for coccyx fracture is successful in about 90% of cases.^[1]^ The duration for spontaneous recovery is usually one to several months.^[7]^ Coccyx fractures typically do not present with neurologic deficits.^[8]^ In our patient, the refractory coccydynia occurred after she returned to work. The muscle forces on the fracture dislocation of the coccyx can slow the rate of healing.^[2,3]^ Hence, it is likely that the cause of her refractory coccydynia was the use of muscle force or her prolonged sitting posture during work. Several alternative and usually more aggressive treatment approaches, such as pelvic floor rehabilitation, intra-rectal manipulation, intra-nasal calcitonin administration, and transcutaneous electrical nerve stimulation, have been reported to improve refractory coccydynia under some circumstances; however, these approaches have only been assessed in small sample sizes, with some being accompanied by discomfort during treatment.^[1]^ If the coccydynia persists or is aggravated after more than 2 months of conservative treatment, an orthopedic consultation is indicated to evaluate the need for local steroid injection or coccygectomy.^[2,3,7]^ However, coccygectomy is associated with a high complication rate and may fail to relieve the coccydynia.^[1]^ Therefore, some patients may try other conservative treatments before making a final decision regarding surgery, as observed in our case.

As mentioned earlier, laser acupuncture has previously been shown to exert anti-inflammatory effects and to promote wound healing, thus alleviating acute or chronic pain.^[4,5]^ A possible mechanism underlying the produced analgesia is suppression of inducible nitric oxide synthase and microglial p-p38, which are cytokines associated with inflammation.^[5]^ To our knowledge, no previous documentation of laser acupuncture or traditional acupuncture applied for coccyx fracture or coccydynia exists. However, it has been demonstrated that acupuncture at *Shenshu* (BL23) decreases paw swelling and the histologic scores of inflammation in the synovial tissue in animal models.^[9]^ Furthermore, acupuncture at *Bai Hui* (GV20) reduces the infiltration of inflammatory cells and improves neurological function in animal models.^[10]^ In humans, acupressure at BL32 has been stated to be effective in relieving labor pain,^[11]^ while acupuncture at BL23, BL25, and BL32 is commonly used to reduce low back pain.^[12]^ Our finding that LLLT at these points reduced refractory coccydynia following coccyx fracture supports the analgesic properties of LLLT, and extends its applicability to patients with coccyx fractures.

According to a PubMed database search we conducted using the terms “low-level laser therapy” and “fracture,” most of the published research utilized animals and revealed that LLLT improved bone healing.^[13–16]^ The several identified human studies demonstrated that LLLT enhanced bone healing in patients with maxillary cystic defects,^[17]^ closed bone fractures in the wrist and hand,^[18]^ and tibia fractures.^[19]^ A recent systematic review likewise showed that LLLT may be beneficial for fracture healing, according to both human and animal studies, with the possible effects of LLLT being exerted by regulating genes associated with inflammation control, increasing osteogenic gene expression, improving bone circulation, and increasing the metabolism of bone callus, which contributed in bone healing in animal models.^[6]^

In sum, the present report demonstrated that laser acupuncture exerted analgesic effects in a patient with refractory coccydynia following a traffic-accident induced coccyx fracture. Optimal analgesic effects were achieved in our patient, as the NRS score decreased from 8 initially, to 2 after 11 weeks of treatment, and finally to 0 after 21 weeks of treatment. Bone healing was also observed in this patient after 11 weeks of treatment. The mechanisms underlying LLLT's analgesic and bone healing effects are likely associated with inflammation control, increasing osteogenic gene expression, improving bone circulation, and increasing the metabolism of bone callus, as suggested previously by animal model studies. The potential effects of LLLT on bone healing and the underlying mechanisms of LLLT's analgesic effects on fracture-related pain, including coccyx fractures, need to be clarified in future clinical studies. Nevertheless, our findings suggest that laser acupuncture is a good conservative treatment option for patients with coccyx fracture.

## Acknowledgments

Authors would like to thank Ms Lana Wood and Mr Paul Wood for the assistance on the refinement of the figures.

## Author contributions

**Conceptualization:** Chun-En Aurea Kuo, Szu-Ying Wu.

**Data curation:** Chien-Hung Lin.

**Investigation:** Chien-Hung Lin.

**Methodology:** Chia-Hung Hung.

**Supervision:** Chun-En Aurea Kuo, Wen-Long Hu.

**Writing – original draft:** Chien-Hung Lin.

**Writing – review & editing:** Chun-En Aurea Kuo, Yu-Chiang Hung.

Chun-En Aurea Kuo orcid: 0000-0002-1563-8651.
